# Pericellular collagen I coating for enhanced homing and chondrogenic differentiation of mesenchymal stem cells in direct intra-articular injection

**DOI:** 10.1186/s13287-018-0916-z

**Published:** 2018-06-27

**Authors:** Hansong Xia, Chi Liang, Pan Luo, Junjie Huang, Jinshen He, Zili Wang, Xu Cao, Cheng Peng, Song Wu

**Affiliations:** 1 Department of Orthopaedics, 3rd Xiangya Hospital, Central South University, Changsha, 410013 China; 2 Department of Burns and Plastic Surgery, 3rd Xiangya Hospital, Central South University, Changsha, 410013 China

**Keywords:** Cartilage injury, Cartilage repair, Chondrogenic differentiation, Bone marrow mesenchymal stem cells, Homing

## Abstract

**Background:**

Direct intra-articular injection (DIAI) of mesenchymal stem cells (MSCs) is a promising technique for cartilage repair. However, the repair process was hindered by the absence of scaffold and poor cell–matrix interactions.

**Methods:**

In this study, we developed a pericellular collagen I coating (PCC) on MSCs. The overall performances of MSC-PCC homing, chondrogenic differentiation, and cartilage regeneration have been comprehensively evaluated in a New Zealand rabbit model. Firstly, we examined the morphology and physical characteristics of PCC. Secondly, MSC ex-vivo cartilage slice adhesion and in-vivo cartilage defect homing were observed using multiscale methods. Thirdly, the precartilage condensation of cell pellets formed by aggregation of MSCs was examined to evaluate the cartilage-inducing potential of PCC. Finally, the cartilage regeneration by DIAI of PCC-coated MSCs was observed and scored macroscopically and histologically.

**Results:**

In general, the cell adhesion and homing assay revealed that PCC facilitated MSC adhesion on cartilage slices, enhancing MSC homing and retention to cartilage defect. This increased homing ratio was accompanied by an increasing cell–cell contact. Compared with naked MSCs, the cell pellets formed by PCC-coated MSCs exhibited more evident appearance of condensation. In pellets, cell–cell interaction has been significantly stimulated, inducing the expression of condensation marker N-cadherin, and subsequent chondrogenic marker collagen II and aggrecan. By 12 weeks after DIAI, cartilage defects have been repaired by MSCs to varying degrees. Overall, PCC significantly enhances the quality of cartilage regeneration judging from macroscopic observation, ICRS score, histological examination, and collagen type I, II, and X immunohistochemical staining.

**Conclusions:**

The capacity and viability of MSCs can be enhanced by collagen I coating, which provides cues for enhancing cell homing and differentiation. Our method provides a novel strategy for stem cell therapy.

**Electronic supplementary material:**

The online version of this article (10.1186/s13287-018-0916-z) contains supplementary material, which is available to authorized users.

## Background

Regeneration of the cartilage surface is essential for preventing osteoarthritis (OA). The combination of mesenchymal stem cells (MSCs), growth factors, and 3D scaffold by cartilage tissue engineering is the most prevalent strategy currently being used [1]. There are many promising methods applying engineered MSCs to cartilage regeneration, yet optimization of the proliferation and chondrogenic differentiation of these MSCs are poorly developed. This has led to the utilization of alternative strategies such as direct intra-articular injection (DIAI) of MSCs.

It has been reported that MSCs home to target lesion sites [2]. Based on this property, these cells may be transplanted into a cartilage defect by a simple and mini-invasive approach. Both animal and clinical studies have confirmed the effectiveness of DIAI in cartilage repair [3, 4]. It is an attractive strategy with a great potential for clinical application. Nevertheless, DIAI faces certain challenges. The low homing ratio and the difficulty in chondrogenic differentiation are the two principal obstacles for DIAI [5]. For example, in a study by Leijs et al. [6], the majority of the injected MSCs located freely in joint space or attached to synovium, while only a small amount of cells homed to cartilage. Stem cell therapies rely on the precise location of cells in the targeted tissue [2]. Low homing ratio limits the repair ability [7]. Unlike tissue engineering where cells are seeded on a 3D scaffold, using DIAI it is difficult to regulate MSC differentiation in the absence of appropriate cell–matrix interaction. Although several efforts have been made to overcome these limitations, no satisfactory results have yet been achieved [6, 8–12].

In cartilage, chondrocytes are encapsulated in a thin rim of matrix termed the pericellular matrix (PCM). This matrix is pivotal in anchoring cells to the cartilage matrix and maintaining chondrogenic differentiation [13, 14]. The PCM and the encapsulated chondrocyte forms the chondron which is the functional unit in cartilage. This structure provides a novel strategy for stem cell therapy. The injected stem cells are coated with a PCM that, in theory, is designed to facilitate cell homing and to induce differentiation. The scaffold should possess good biocompatibility, low immunogenicity, and, most importantly, a simple preparation procedure for clinical application.

In light of these factors, type I collagen (col. I) seems to be a promising candidate. In the early stage of chondrogenesis, MSCs reside in the stem cell niche abundant in col. I, which provides essential cues for MSC condensation and subsequent chondrogenic differentiation [15, 16]. Col. I has a high affinity for fibronectin (FN) that is highly expressed at lesion sites and may anchor cells to cartilage [17]. This anchoring effect is recognized as a potent initial factor in cell adhesion [18]. In this study, we established a pericellular collagen I coating (PCC) by a simple coincubation procedure and achieved significant effects on allogeneic MSC homing, chondrogenic differentiation, and cartilage regeneration.

## Methods

### MSC isolation and culture

All animal experiments were conducted according to the approved animal protocol of the Third Xiangya Hospital of Central South University. The New Zealand rabbits were given 1 week to become acclimated to laboratory conditions. Surgery was performed under general anesthesia, and all efforts were made to minimize animal suffering. MSCs were isolated from bone marrow (BM) of the tibia and femur of New Zealand rabbits (1 month old, male). BM was harvested and washed with PBS (5 min, at 200 × *g*). Cells were cultured in DMEM (Gibco, USA) supplemented with 10% FBS (Gibco, USA) at 5% CO_2_, 95% humidity, and 37 °C. Nonadherent cells were removed carefully after 24 h of incubation. Thereafter, the medium was replaced every 3 days. Cells were subcultured when 80% confluence was reached.

### MSC identification

Isolated primary MSCs were identified by morphology, surface biomarkers, and their ability for multiple differentiation. The morphological appearance of cultured MSCs was observed daily by inverted microscope (Nikon, Japan).

For CD antigen analysis, MSC suspension was blocked with 1% BSA for 20 min and incubated with anti-rabbit CD11b, CD34, CD45, CD90, and Vimentin antibodies (Abcam, UK) and isotype control antibodies for 20 min at room temperature. Then, cells were washed and analyzed using flow cytometry (BD FACSCalibur, USA).

The Oricell rabbit MSC differentiation media (Cyagen, USA) were utilized to induce MSC multiple differentiation. For adipogenic differentiation, MSCs were cultured in adipogenic differentiation medium A (induction medium) for 3 days and in medium B (maintenance medium) for 1 day. After four cycles, MSCs were cultured in medium B for 7 days before Oil Red O stain. For osteogenic differentiation, MSCs were culture in osteogenic differentiation medium for 4 weeks and subsequently stained by Alizarin Red. For chondrogenic differentiation, MSCs were centrifuged to form a cell pellet and cultured in chondrogenic differentiation medium for 2 weeks. Pellets were formalin fixed for Alcian blue stain.

### MSC labeling

MSCs were dual-labeled by bioluminescent and fluorescent signals. MSCs were infected with lentivirus-expressing luciferase (Genechem, Shanghai, China) for 8 h and then cultured in DMEM with 10% FBS for 40 h. Subsequently, cells were labeled with 0.1% CM-DiI (Thermo Fisher, USA) at 37 °C for 5 min and then at 4 °C for 15 min. Dual-labeling was examined by the bioluminescent imaging system (BLIS; PerkinElmer, USA) and fluorescent microscope (Olympus, Japan) respectively.

### Pericellular collagen I coating on MSCs

Col. I from rat tail (3 mg/ml; Life Technologies, USA) was diluted to 100 μg/ml in DMEM with 15% FBS. Then 2.5 × 10^5^ adherent MSCs (passage 3) were digested with trypsin and washed with PBS and resuspended in 600 μl of diluted col. I solution. To form PCC, the MSC–col. I suspension was incubated in an ultralow-attachment culture plate (Corning, USA) at 5% CO_2_, 37 °C for 2 h. The cells were then carefully washed five times with PBS (at 100 × *g* for 5 min) to remove the unconjugated col. I. The concentration of residual col. I in the suspension was quite low (Additional file 1: Figure S1). To observe PCC, cells were fixed with 4% PFA, blocked with 2% BSA (Gibco, USA), and incubated with 1.5 μg/ml anti-col. I (Abcam, UK) for 2 h at 20 °C and 1 μg/ml anti-mouse IgG (H + L) conjugated to FITC (Abcam, UK) for 30 min at 20 °C. The nucleus was stained with DAPI (Boster, China). The coating ratio was calculated using a fluorescent microscope (Olympus, Japan) from 10 low-power fields. Cells were viewed using a laser scanning confocal microscope (LSCM; Olympus, Japan).

The ultrastructure of the PCC was investigated by scanning electron microscope (SEM) (JSM-6400F; JEOL, Japan). The MSCs coated by col. I were washed with 0.1 M sodium phosphate buffer (pH 7.2) three times, then fixed in 2.5% glutaraldehyde overnight, and post-fixed in 1% osmium tetroxide for 1 h, dehydrated in ethanol of gradient concentration. Dried samples were coated with gold via a sputter-coater at ambient temperature. Micrographs were taken by SEM.

We used an atomic force microscope (AFM) (Nano Wizard II; JPK, Germany) to detect the force–distance curve of MSCs and MSCs-PCC (*n* = 6 for each group) individually, and calculated Young’s modulus using the Hertz model:

$$ \boldsymbol{F}={\delta}^2\frac{\mathbf{2}}{\boldsymbol{\pi}}\frac{\boldsymbol{E}}{\left(\mathbf{1}-{\boldsymbol{\upsilon}}^{\mathbf{2}}\right)}\boldsymbol{\tan}\left(\boldsymbol{\alpha} \right), $$

where *F* represents the indentation force and *δ* represents the indentation depth, both obtained from the force–distance curve; *υ* represents Poisson’s ratio, α represents the half open-angle, and *E* represents Young’s modulus.

The half open-angle of the probe was 17.5 ± 0.5°, and Poisson’s ratio was 0.5.

### Ex-vivo adhesion assay

The MSC ex-vivo adhesion assay was performed on cartilage slices. The knee joint was exposed by the anteromedial approach. Subsequently, the anterior and posterior cruciate ligaments were transected, and the medial meniscus was excised. The tibial plateau was dislocated forward and fully exposed. Then, the superficial and part of the transitional layer of the tibial plateau cartilage were removed using a 0.5-mm-height blade under the microscope. Three days after surgery, the rabbits were sacrificed and the slices of lesion surface (6 mm in diameter, 100 μm in thickness) were obtained using a 6-mm punch and microtome (Leica, Germany). Slices were put on the bottom of 96-well plates with the lesion surface upward for cell adhesion assay. Then 5 × 10^4^ luciferase/CM-DiI-labeled MSCs, with (MSCs-PCC) or without PCC (naked MSCs), were resuspended in 100 μl DMEM (with 15% FBS), added onto the slices, and incubated for 3 h at 5% CO_2_, 37 °C. Naked MSCs suspended in 10 ng/ml, 500 ng/ml, and 10 μg/ml col. I solution were also tested.

For further analyses, MSCs were treated with 0.2 mg/ml collagenase (type I; Sigma, USA) for 20 min and/or 10 μg/ml anti-integrin β1 (Abcam, UK) for 15 min before adhesion assay, to eliminate the PCC and integrin β1 (ITG β1) respectively. After 3 h of incubation, slices were rinsed with PBS carefully to remove nonadherent cells. For multiscale observation, slices were observed under the BLIS, fluorescent microscope, and LSCM.

### In-vivo homing and retention

An 8 mm × 2 mm full-thickness cartilage defect was made at the medial side of the femoral trochlear groove of 1-month-old rabbits. We first used a blade to draw a rectangular boundary on the relatively flat medial side of the femoral groove, and then manipulated a 2-mm-width blade to scoop the cartilage to expose the calcified cartilage layer. Three days after surgery, 3 × 10^5^ luciferase/CM-DiI-labeled MSCs-PCC or naked MSCs were resuspended in 500 μl DMEM and injected into the knee joint. MSCs suspended in col. I solution were also investigated. Rabbits were free for movement after injection. On day 2, 7, and 14 after injection (*n* = 3 joints in each group for a time point), the joint was opened (the bilateral of quadriceps cut open and flipped to distal; Fig. 4a) to expose the articular surface and rinsed with PBS to remove the nonadherent cells. Cell homing and distribution was observed directly under the BLIS and by fluorescent microscope. Cell homing was calculated by the total radiance within the cartilage defect region by 2 days and retention was calculated by the ratio of this radiance at 7 days or 14 days to that at 2 days. The frozen sections of the 2-day samples were made to observe the attached MSCs using a fluorescence microscope.

### Cell pellet condensation and chondrogenic differentiation

After PCC, 10^7^ MSCs in 5 ml DMEM (with 10% FBS) were centrifuged to form a cell pellet, and incubated at 37 °C for 18 h, 48 h, and 14 days. Naked MSCs were used as control. Pellets formed by naked MSCs and incubated in chondrogenic differentiation medium (Cyagen, USA) were used as positive control for differentiation assessment.

At 18 h, pellet lysates were probed with mouse primary antibodies (Boster, China) against N-cadherin and tubulin overnight at 4 °C. The blots were incubated with secondary antibodies (Boster, China) for 1 h at room temperature. At 48 h, pellets were fixed with formalin, dehydrated with ethanol, split to expose the core area, and observed under a SEM (JSM-6400F; JEOL, Japan). On day 14, pellets were harvested and total RNA was extracted using the E.Z.N.A. Total RNA Kit II (Omega Bio-tek, USA) following the manufacturer’s protocol. Reverse transcription reactions were performed using ReverTra Ace qPCR RT Master Mix (TOYOBO, Japan). RT-qPCR reactions were conducted using THUNDERBIRD SYBR qPCR Mix (TOYOBO, Japan). The initial denaturation was 95 °C for 60 s, and 40 cycles of denaturation at 95 °C for 15 s, annealing at 60 °C for 30 s, and extension at 72 °C for 60 s. The primer sequences for this study were as follows: GAPDH, forward 5′-GAACATCATCCCTGCCTCCACTG-3′ and reverse 5′-ATGCCTGCTTCACCACCTTCTTG-3′; col. I, forward 5′-GGCAACAGCAGGTTCACTTA-3′ and reverse 5′-GGCAAACGAGATGGCTTATT-3′; col. II, forward 5′-GGGTGGACATAGGGCCCGTCTG-3′ and reverse 5′-CTTGCTTCTGGGCGGGGCGTTG-3′; aggrecan, forward 5′-AGGTCTCGCTGCCCAACTA-3′ and reverse 5′-GTAGCCTCGCTGTCCTCAAG-3′; and SOX-9, forward 5′-GGGAAGCTCTGGAGACTGCT-3′ and reverse 5′-TGTAGTCCGGGTGGTCTTTC-3′. The transcriptional level of the target genes, normalized to GAPDH, and the fold-change difference were calculated by lg^2–△△Ct^, with reference to the naked BMSCs.

### Histological and immunohistochemical evaluation

Cartilage defect was made as described earlier, using 3-month-old rabbits. MSCs-PCC (10^6^ cells) in 500 μl DMEM were injected into the knee joint on day 7 after operation. Rabbits were sacrificed 6 and 12 weeks later (*n* = 4 joints in each group for one time point). The cartilage regeneration was evaluated and compared using ICRS scores [19] macroscopically and Wakitani scores [20] histologically. Anti-col. I, anti-col. II (Thermo Fisher, USA), and anti-col. X (Abcam, UK) antibodies were used for immunohistochemistry of the repaired tissues.

### Statistics

Values are expressed as the mean ± SD in the text and figures unless otherwise noted. Statistical significance was determined by Student’s *t* test or analysis of variance (ANOVA) using SPSS 17.0 (SPSS, Inc., Chicago, IL, USA). *P* < 0.05 was considered statistically significant.

## Results

### MSC identification

**Fig. 1:**
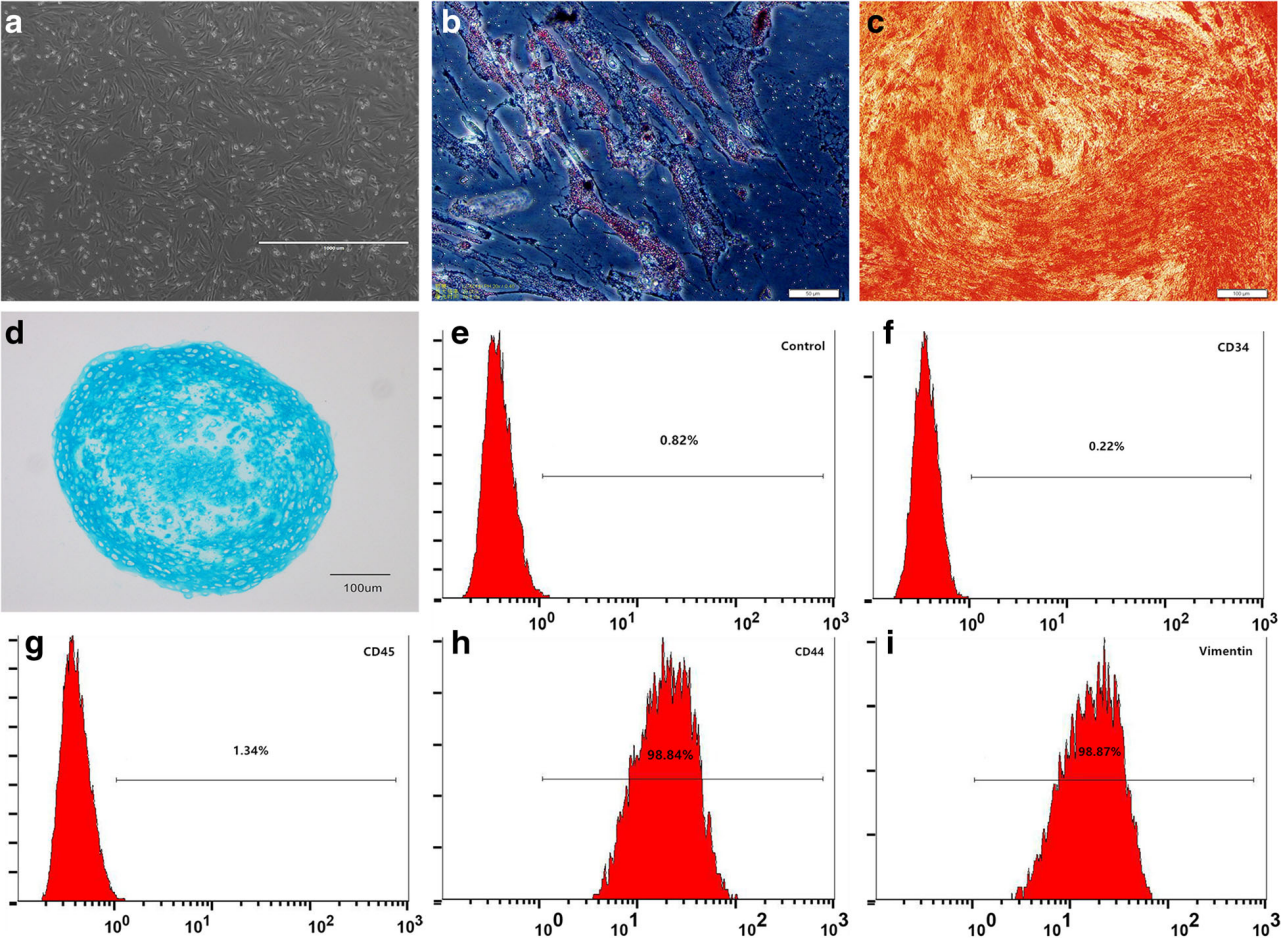
MSC identification. **a** Fibroblast-like cells adhered to culture surface at 3 days after seeding, confluence about 70% (scale bar, 1000 μm). **b** Alizarin Red staining of osteogenesis induction (scale bar, 100 μm). **c** Oil Red O staining of adipogenesis induction (scale bar, 50 μm). **d** Alcian Blue staining of chondrogenesis induction (scale bar, 100 μm). **e**–**i** Mean percentages of New Zealand rabbit MSCs labeled with isotype control IgG, CD34, CD44, CD45, and Vimentin, determined by flow cytometry

At day 7 of primary culture, the MSCs reached 80% confluency and formed many colony-forming unit fibroblasts (CFU-F). Most of the cells exhibited typical spindle shape while some were triangular or irregular (Fig. 1a). Isolated cells displayed adipogenic, osteogenic, and chondrogenic differentiation capacity, respectively (Fig. 1b–d). Surface antigen analysis showed that the cells expressed mesenchymal markers CD44 (98.84%) and Vimentin (98.87%) but poorly expressed hematopoietic markers CD34 (0.22%) and CD45 (1.34%) (Fig. 1e–i).

### Immunofluorescence staining, ultrastructure, and physical characteristic of PCC on MSCs

**Fig. 2:**
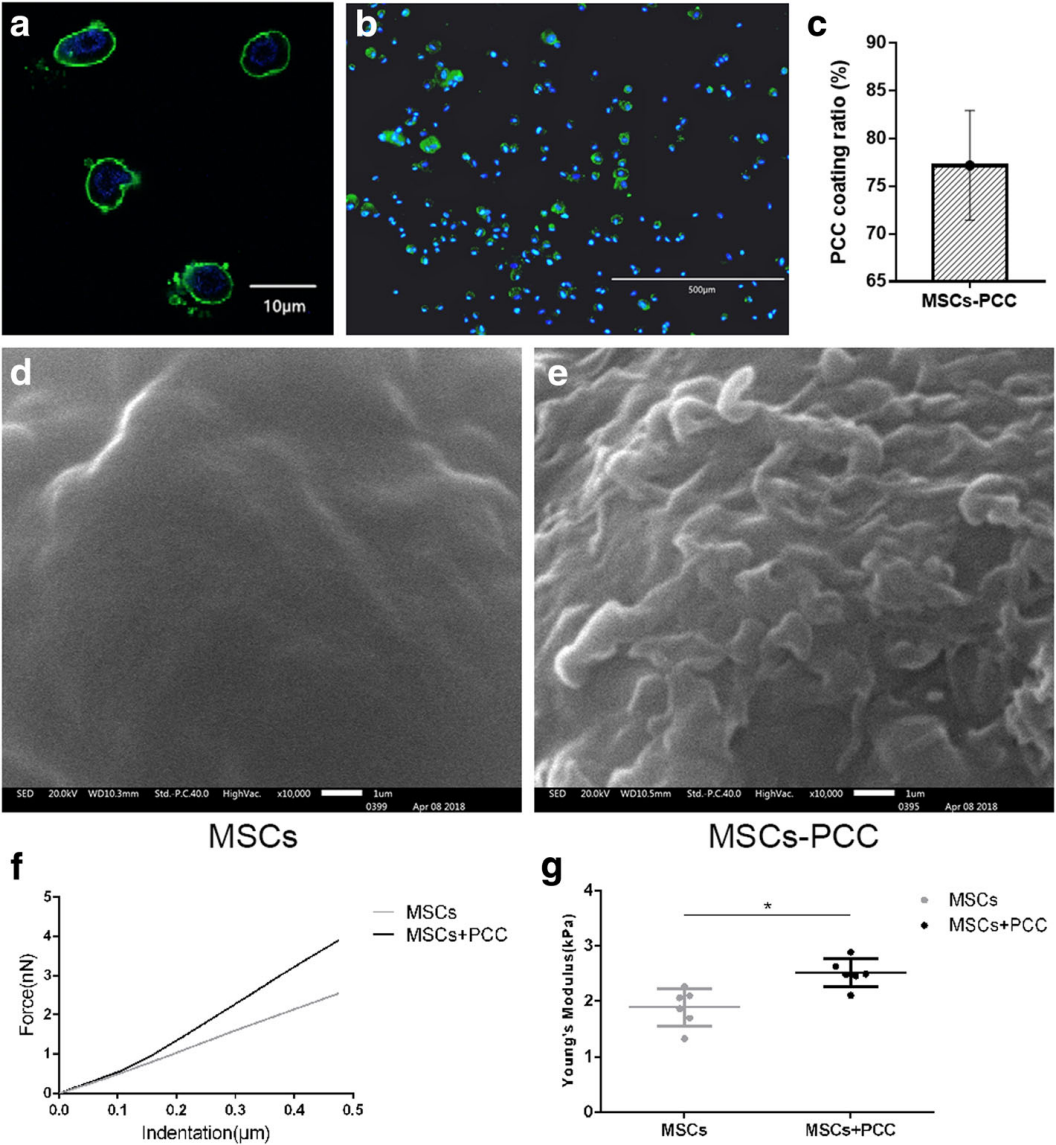
Pericellular collagen I coating. **a, b** MSCs coated with PCC observed by laser confocal microscopy (**a** scale bar, 10 μm) and fluorescence microscope (**b** scale bar, 500 μm). PCC stained by FITC-labeled anti-col. I antibody green, nucleus stained by DAPI blue. **c** Coating ratio calculated by fluorescence microscopy (*n* = 5). **d** SEM image of single MSC under 10,000× magnification. **e** SEM image of single MSC coated by col. I under 10,000× magnification. **f** Force–distance curve of MSCs and MSCs-PCC groups. **g** Young’s modulus of MSCs and MSCs-PCC groups. MSC mesenchymal stem cell, PCC pericellular collagen I coating. *p < 0.05

After 2 h of coincubation, cells coated with PCC were visualized by immunofluorescence using FITC-labeled antibody under the LSCM. In cross-section of the cells, col. I was detected at the pericellular region, forming a thin membranous and fragmented scaffold (Fig. 2a, b). Cells were nonuniformly coated, as some were attached by massive col. I while some had no fluorescent signal. In general, PCC was detected around the majority of cells. The coating ratio calculated from 10 low-power fields was about 77% (Fig. 2c).

The SEM observation results showed that the single cell surface of the MSCs-PCC group (Fig. 2d) was more uneven than those not coated by col. I (Fig. 2e). Based on the force–distance curve (Fig. 2f), Young’s modulus of the MSCs-PCC group was higher than that of the MSCs group, 1.89 ± 0.31 and 2.51 ± 0.23, respectively (Fig. 2g).

### Ex-vivo adhesion

**Fig. 3:**
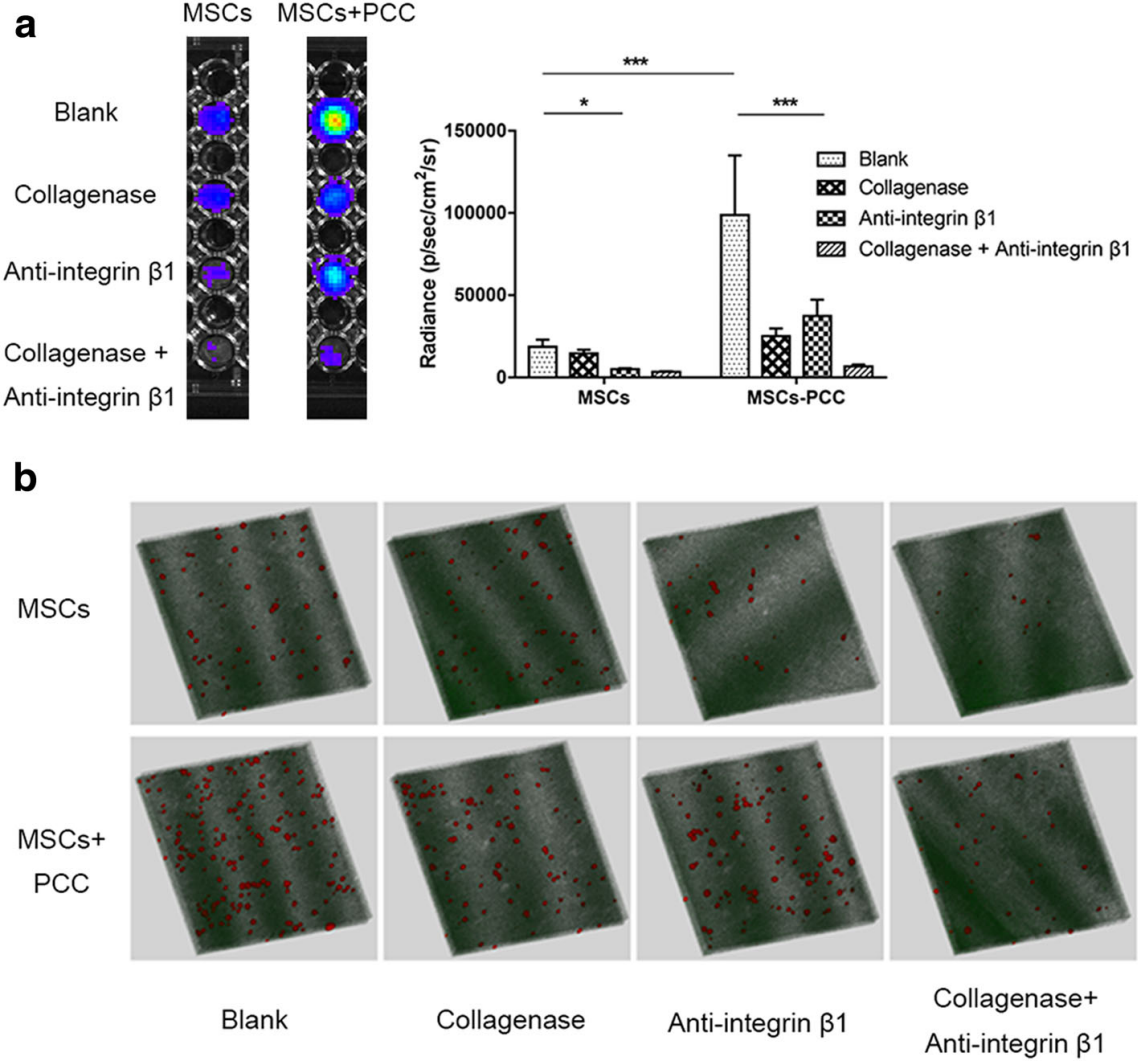
Ex-vivo adhesion. **a** BLIS analysis of MSCs adhered on cartilage slices. MSCs pretransfected by luciferase and total adhered cell number calculated by luminescent intensity. MSCs and MSCs-PCC treated with collagenase, anti-integrin β1, and both before adhesion assay (*n* = 3 in each group). *p < 0.05, ***p < 0.01. **b** Representative laser confocal images showing adherent CM-DiI-labeled MSCs on surface of cartilage slices (*n* = 3 in each group). MSC mesenchymal stem cell, PCC pericellular collagen I coating

To study the effect of PCC on MSC cartilage adhesion, we incubated luciferase/CM-DiI dual-labeled MSCs on cartilage slices. Adhered cells were observed by multiscale methods. Using the BLIS, the cell number on the whole cartilage slice was counted. Compared to naked MSCs, PCC significantly enhanced MSC adhesion on cartilage, with an average 5-fold increase in luciferase signal (Fig. 3a). As a control, the MSCs suspended in col. I solution did not enhance the ability of MSC adhesion on the cartilage; rather, the radiance decreased in the 10 μg/ml col. I solution group (Additional file 2: Figure S2). Furthermore, cell adhesion was measured after eliminating PCC and ITG β1. For naked MSCs, as expected, blocking of ITG β1 prevented the adhesion of the majority of cells, whereas collagenase had minimal effect (Fig. 3a). In contrast, in MSCs-PCC the increase in adhesion arising from PCC was abolished by collagenase. Interestingly, this adhesion-promoting effect was also hampered significantly by ITG β1 blocking. Moreover, a more dramatic effect was observed when collagenase and anti-ITG β1 were combined (Fig. 3a). This indicates a synergistic mechanism between PCC and ITG β1.

LSCM analysis revealed similar results with the BLIS. The cell density on slices of the MSCs-PCC group was higher than that of the naked group. Additionally, more cell-to-cell contact was observed with increasing density. There was no difference in cell morphology between the groups (Fig. 3b).

### In-vivo homing and retention

**Fig. 4:**
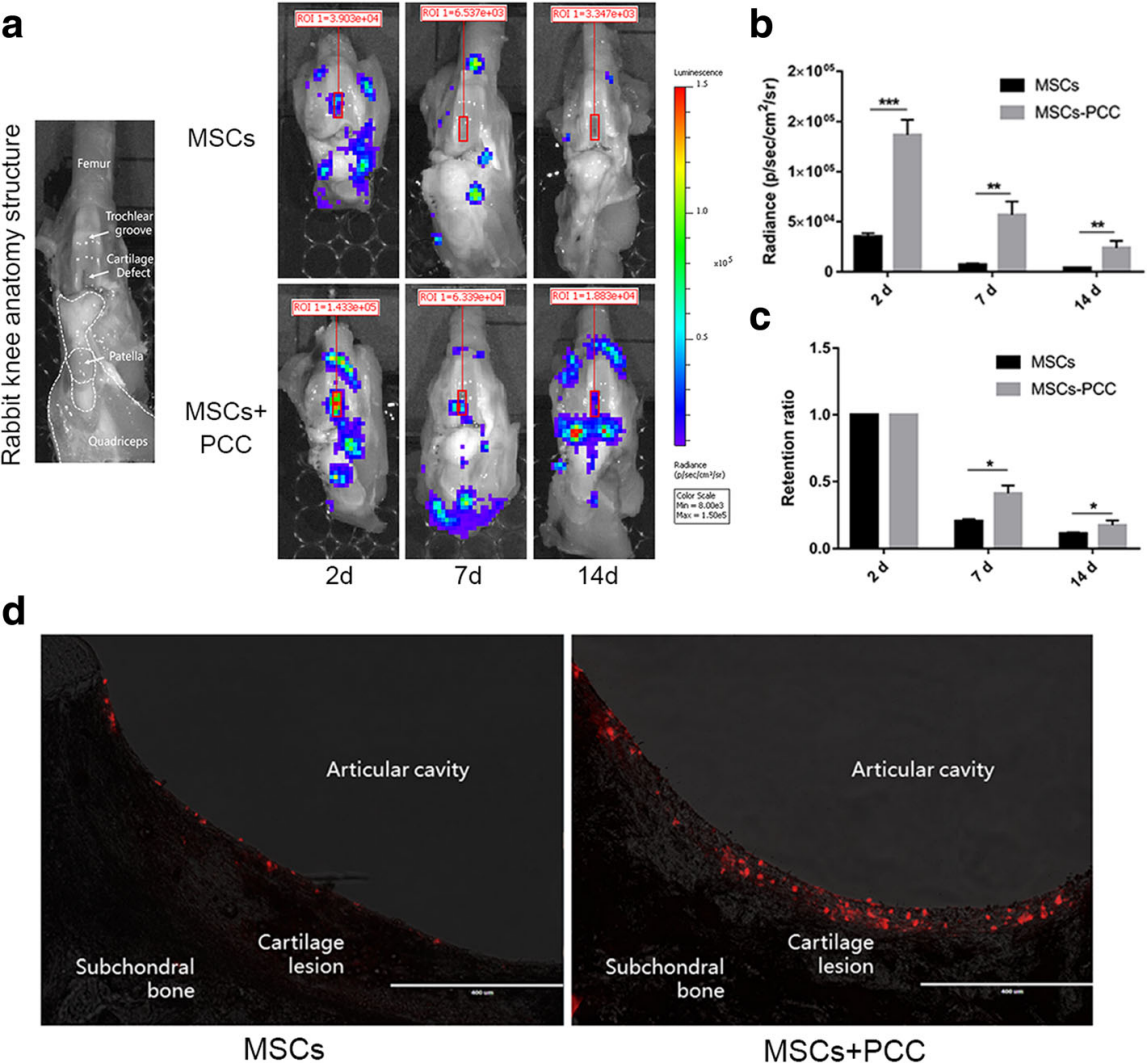
In-vivo homing and retention. **a, b** BLIS analysis of luminescent distribution within left knees of New Zealand rabbits at 2, 7, and 14 days after DIAI of luciferase-labeled MSCs or MSCs-PCC (**a** *n* = 3 in each group). Red frames indicate cartilage defect. Luminescent intensity of total ROI radiance within cartilage defect (red frame) calculated to reveal homing of MSCs (**b**). **p < 0.01 and ***p < 0.001. **c** Retention of MSCs within cartilage defect calculated by ratio of radiance at 7 days or 14 days to that at 2 days in (**b**) *p < 0.01. **d** Sagittal frozen sections of cartilage lesion site, red particles are CM-DiI-labeled MSCs (scale bar, 400 μm). d days, MSC mesenchymal stem cell, PCC pericellular collagen I coating, ROI region of interest

MSC homing and distribution within the joint was observed using the BLIS. In general, after DIAI, cells gathered at the recess of suprapatellar bursa and the peripheral rim of meniscus. MSCs in all groups displayed varying degrees of homing toward cartilage lesion. Compared to naked MSCs, MSCs-PCC exhibited marked enhancement of cell adhesion and viability in the whole joint (Fig. 4a). Specifically, we found that PCC significantly promoted MSCs homing with 4-fold increase in the total ROI radiance within the lesion site (Fig. 4b). This was further confirmed with fluorescence microscopy where a large number of MSCs-PCC gathered on the lesion surface, with higher cell density and more cell–cell interaction. In comparison, naked MSCs were scattered sparsely (Fig. 4d). As a control, mere incubation in col. I solution failed to increase the number of MSCs homing to the surface of injured cartilage (Additional file 3: Figure S3a–c). Cell retention in the lesion site was also observed. Within 14 days after DIAI, the cell viability decreased dramatically in both groups. We found that PCC supported cell retention and survival in the lesion site (retention ratio increased 2-fold at 7 days and 1.5-fold at 14 days; Fig. 4c). It remains to be determined whether this was due to the direct effect of PCC or higher cell density and supportive cell–cell interaction.

### Condensation and chondrogenic differentiation

**Fig. 5:**
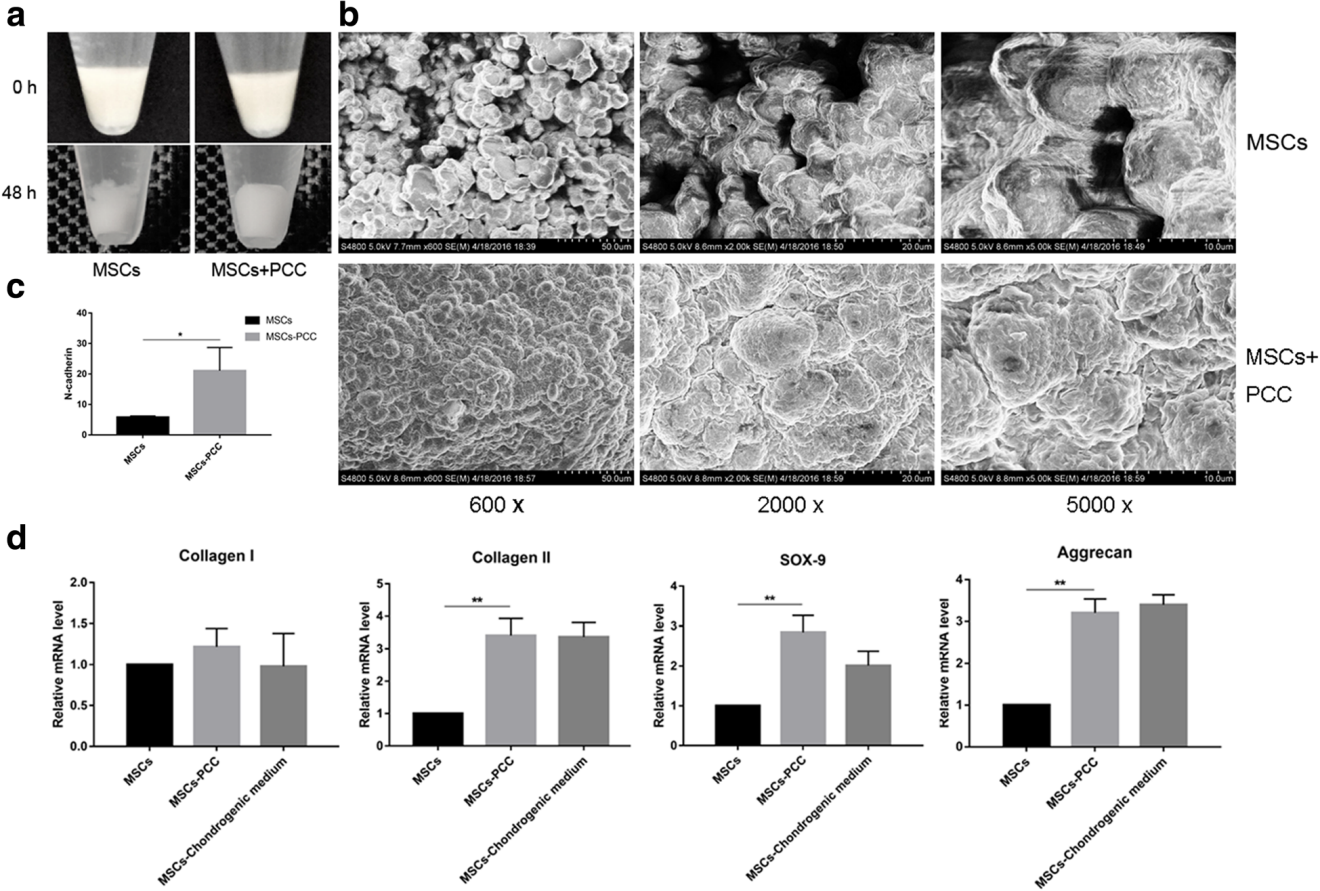
Condensation and chondrogenic differentiation. **a** Macroscopic view of MSC and MSC-PCC pellet groups cultured in 1-cm centrifuge tube at 0 h and 48 h. **b** SEM images of cell pellets under 600×, 2000×, and 5000× magnification. **c**. Expression levels of N-cadherin in MSC and MSC-PCC pellet groups cultured after 18 h (*n* = 3 in each group). *p < 0.05. **d** RT-qPCR of col. I, col. II, SOX-9, and aggrecan in MSC and MSC-PCC pellets cultured after 14 days (*n* = 3 in each group). MSC mesenchymal stem cell, PCC pericellular collagen I coating. **p < 0.01

The precartilage condensation of MSCs was examined to evaluate the cartilage-inducing potential of PCC. Macroscopically, the pellets of MSCs-PCC contracted by 48 h of incubation, whereas naked MSC pellets still remained flocculent (Fig. 5a). When the core of the pellets was observed under the SEM, cells displayed round shape morphology. No remarkable effect of PCC on MSC morphology was observed, except that MSCs-PCC looked slightly irregular in shape. However, PCC significantly stimulated cell–cell interaction. Under 600×, 2000×, and 5000× magnification, we found that MSCs-PCC interacted with each other much more tightly with almost no gap. In contrast, control cells adhered loosely and apparent space in the pellets was detected (Fig. 5b). Moreover, the protein level of N-cadherin, a condensation marker, was elevated in MSC-PCC pellets compared to naked MSC pellets (3.6-fold increase; Fig. 5c).

The chondrogenic markers in pellets were examined after 14 days of incubation. MSC pellets incubated with chondrogenic medium were used as positive controls. PCC significantly induced MSC chondrogenic differentiation with a mean of 2.8-fold increase in SOX-9, 3.4-fold increase in col. II, and 3.2-fold increase in aggrecan expression compared to naked MSCs (Fig. 5d). The expression of fibrotic marker col. I was not affected.

### Macroscopic observation and assessment

**Fig. 6:**
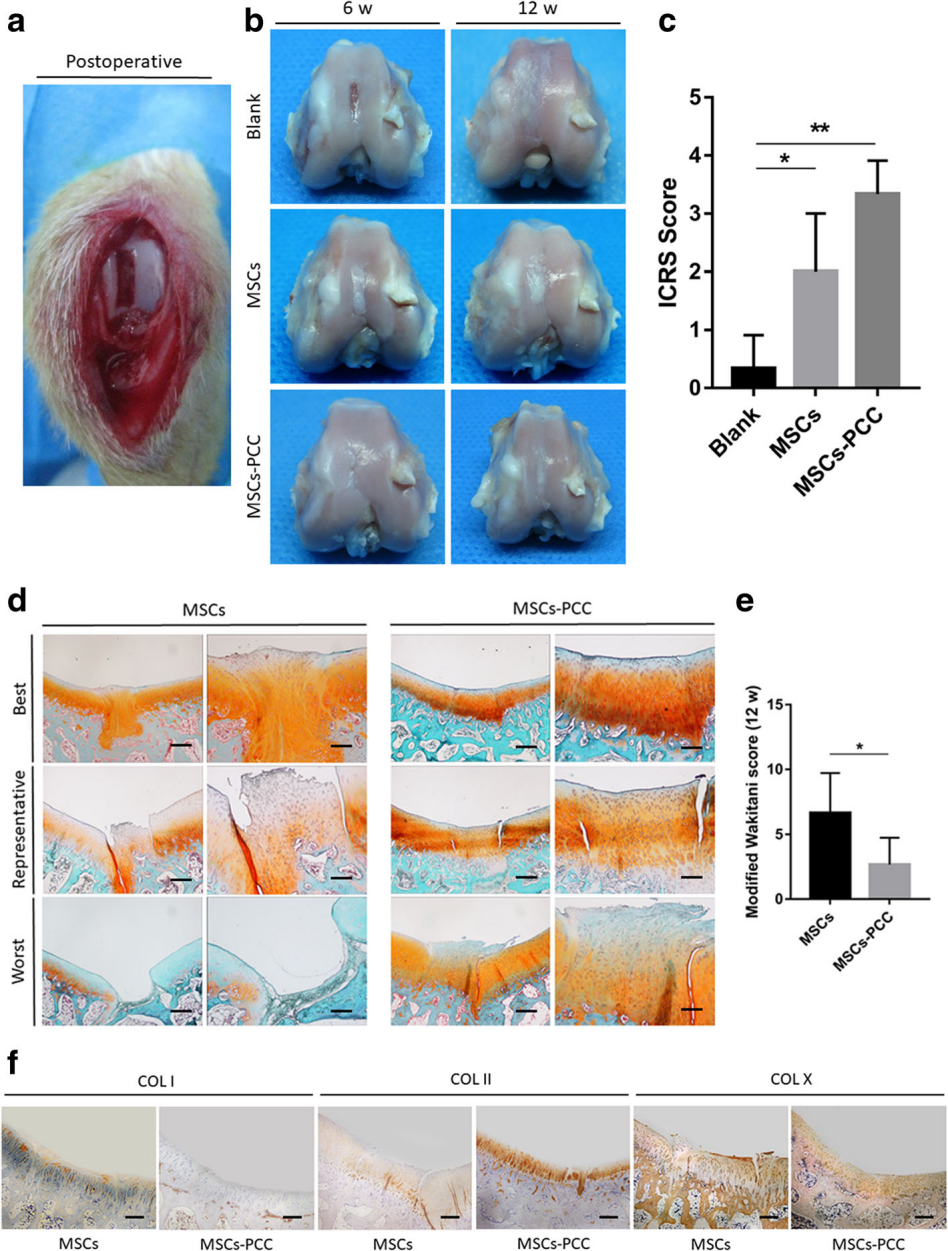
Macroscopic observation and histological scoring. **a**. Macroscopic observation of postoperative cartilage defects. **b** Macroscopic observation of cartilage defects after cell transplantation at 6 and 12 weeks (*n* = 4 in each group). **c**. ICRS score of blank, MSCs, and MSCs-PCC groups after injection at 12 weeks. **d** Safranin O-Fast Green staining (scale bars of 4× and 10× images, 100 μm and 40 μm respectively; *n* = 4 in each group). **e** Modified Wakitani score of MSCs and MSCs-PCC groups after injection at 12 weeks (*P* = 0.0202, *n* = 4 in each group). **f** Immunohistochemical expression of col. I, col. II, and col. X in full-thickness chondral defects in trochlear groove at 12 weeks after injection of MSCs and MSCs-PCC (scale bar, 100 μm; *n* = 4 in each group)

No signs of infection, cell rejection, or abnormal inflammatory reaction was observed after transplantation. Rabbits were sacrificed at 6 weeks and 12 weeks. No obvious synovial proliferation, osteophytes, and tumor formation was observed. At 6 weeks, the defects were still clearly visible in the nontreated group (blank), and spontaneous repair occurred to some extent. At 12 weeks, this evolved into severe cartilage degeneration and apparent fibrillation (Fig. 6b).

In the MSCs group, the defects were almost completely filled by fibrous like tissue at 6 weeks and the periphery border remained clear. At 12 weeks, local degeneration and fibrillation still occurred at the original defect area (Fig. 6b). In the MSCs-PCC group, defects were filled by hyaline cartilage-like tissue and the border was slightly indiscernible. Although some fibrous tissue still existed, no obvious degeneration was observed for up to 12 weeks (Fig. 6b).

The ICRS scoring system was used to assess the repair results. Overall, significant difference was found between the MSCs group (score = 1.67 ± 0.5774) and the blank group (score = 0.33 ± 0.5774, *P* = 0.0474). PCC significantly enhanced cartilage repair quality compared to the MSCs group (score = 3.33 ± 0.5774, *P* = 0.0241; Fig. 6c).

### Histological examination scoring

In the MSCs group, we observed varying degrees of cartilage regeneration within the defect sites. Most defects were incompletely filled with an irregular and sunken surface and apparent gaps at the lateral interface. Some defects were filled by regenerative cartilage with abnormal tissue structure and cell morphology. Decreased matrix Safranin O staining and a layer of fibrous regeneration were also observed (Fig. 6d). In contrast, most defects in the MSCs-PCC group were filled by hyaline cartilage-like tissue with acceptable surface regularity, cell morphology, and matrix staining. The lateral integration was still unsatisfactory in some defects, and the degeneration and fibrillation were also found at the superficial region of some samples (Fig. 6d). Nevertheless, the modified Wakitani score for histological evaluation of cartilage regeneration was significantly better in the MSCs-PCC group (score = 2.67 ± 2.082) than in the MSCs group (score = 6.67 ± 3.055, *P* = 0.0202; Fig. 6e).

Immunohistochemical staining for col. II revealed more intense staining in the MSCs-PCC group compared to the sparse and weak staining in the MSCs group. Both groups revealed no apparent difference in col. X staining (Fig. 6f).

## Discussion

The efficient delivery, retention, and chondrogenic differentiation of transplanted cells are major concerns in cell-based therapies of cartilage regeneration [6]. Currently, arthroscopic surgery is the most popular minimally invasive strategy for targeted delivery of cells [21]. Nevertheless, there is a need for a nonsurgical approach. DIAI, due to its admirable simplicity and effectiveness, is a promising nonsurgical method for cartilage regeneration [3, 4].

It is reported that MSCs target and adhere to the cartilage lesion sites after injection [3]. This homing ability of MSCs is the foundation of DIAI. However, the homing ratio remains unsatisfactory with the current methodology [7]. Many efforts have been focusing on different aspects to improve the ratio, including cell sources [9], the intraarticular microenvironment [5, 10–12], and the binding matrix on the cartilage surface [8]. Lee et al. [8] hydrolyzed the proteoglycan-rich matrix on the cartilage surface and exposed the underlying FN to enhance MSC adhesion. Attention has also been focused on synovial microenvironment. For example, magnesium was found to regulate integrin (ITG) expression on the cell surface to facilitate adhesion on cartilage lesion [10, 11]. Hyaluronic acid (HA) in the synovial fluid also exerts a different impact on MSC homing [12]. Leijs et al. [6] reported the effect of an inflammatory and hypoxic environment within the joint. In addition, magnetic particles were utilized to provide homing guidance [22]. These efforts have achieved varying degrees of effects. However, in terms of clinical application, the modification of transplanted cells, as opposed to the synovial environment or cartilage surface, seems to be a more feasible strategy.

Cohen et al. [18] pointed out that, under certain conditions, cell adhesion is initiated by the interaction between a PCM coat and relevant components on the substrate. This interaction subsequently triggers the approach of the cell membrane to the substrate and serves as a prerequisite to ITG-based binding. Cell adhesion is mainly mediated by ITGs. Kinetically, this ITG-based binding is hard to establish, which usually requires minutes to occur. Moreover, ITGs are highly specific to their molecular counterparts. For example, FN that covers the cartilage lesion surface is critical for cell adhesion [8], but the molecular orientation and distance are strict for stable ITG binding [18]. Moreover, the expression of FN-specific ITG (such as ITG α5β1) on undifferentiated MSCs is quite limited [23]. In this situation, the col. I coating could provide a “bridging effect”, before the establishment of ITG binding. The col. I coated on MSCs has a high affinity with FN, and their binding is quicker (less than 1 s) and more adaptable with relatively low specificity [17]. The col. I coating will create more opportunities for ITG binding and cell adhesion. However, this bridging effect is transient and takes place mainly in the early decision-making stage of cell adhesion, whereas firm adhesion still relies on the focal adhesions established by ITGs [24], which offers a partial explanation for the results of our ex-vivo adhesion assay that MSC adhesion improved by col. I still exhibited ITG dependence.

Various methods are used to investigate cell distribution after DIAI [6, 25, 26]. In this study we applied multiscale methods and found that the MSCs gathered at the recess of suprapatellar bursa and the peripheral rim of the meniscus. PCC enhanced cell homing which is consistent with the ex-vivo adhesion results. Prolonged observation revealed a marked decrease in living cell signal. This was probably caused by cell detachment or cell death. The majority of cells injected in suspension died as a result of the severe microenvironment and loss of matrix attachments, which was termed homelessness. The PCM is believed to provide a crucial protective effect for cells in suspension [14]. This corroborates our results that PCC significantly supported cell retention and viability.

Chondrogenic differentiation is another pivotal issue in cartilage regeneration. One process that has been highlighted recently is condensation, which emphasized the key role of the extracellular matrix (ECM) during chondrogenesis [16]. Col. I is highly expressed in fibrocartilage cells and is a critical component in its matrix [27]. As for hyaline cartilage, col. II is the principal component of microfibril in the matrix, while slight col. VI is located in the pericellular region and col. X in the calcified layer [13]. There is hardly any col. I in mature hyaline cartilage. However, during development of cartilage, MSCs firstly reside within the precartilaginous matrix rich in col. I, which interacts with MSCs and provides cues for the subsequent precartilage condensation and chondrogenesis [16]. This is a prerequisite for MSC chondrogenic differentiation [28]. In this process, the characteristic col. I and FN matrix interact with receptors on the cell surface and provide differentiating signals [16]. Based on this, several studies have utilized a col. I scaffold [15], as well as a matrix abundant in col. I [28], to mimic the stem cell niche microenvironment and to induce condensation. But in DIAI, cells are transplanted without the scaffold support. Lee et al. [8] found that homed cells did not exhibit chondrogenic differentiation but developed into fibrocartilage.

In this study, MSC condensation and chondrogenesis were investigated in cell pellets. Significant contraction of the cell pellet was viewed by day 2 in cells coated with PCC, accompanied by enhanced expression of chondrogenic markers SOX-9, col. II, and aggrecan. In the early stages of chondrogenesis, prechondrogenic mesenchymal cells reside in the ECM that is rich in col. I. This col. I provides cues for and facilitates the formation of condensed cell aggregates, accompanied by changes in cell shape and subsequent chondrogenic differentiation. The exact mechanism of this condensation process remains unclear. Raghothaman et al. [15] reported that col. I binds to MSC surface integrins through the activated cell focal adhesion kinase (FAK), which induces upregulation of cell–cell adhesion protein like N-cadherin. On the other hand, col. I changes the shape of cells by mediating the traction between cells, shortening the distance between cells, and increasing the direct contact between cells [15]. These effects generate extensive intercellular connection via N-cadherin and N-CAM, which leads to the expression of chondrogenic transcription factor SOX-9 and cell differentiation [15, 28, 29].

Comparing the repair results, MSCs-PCC revealed superior cartilage regeneration potential and delayed OA development compared with naked MSCs and nontreated groups. However, our study has limitations. Firstly, the lateral integration of regenerated tissue was poor and part of the knees still manifested varying degrees of OA appearance. Moreover, the differentiation phenotype maintenance was not improved and signs of cartilage hypertrophy and ossification were still observed in our neocartilage. Further studies should focus on these points by modifying the composition of this pericellular coating and conjugating various active cytokines.

## Conclusions

PCC facilitated the homing of MSCs to the full-thickness cartilage lesion in a rabbit model and their differentiation toward the chondrocytes, which is beneficial for the regeneration of cartilaginous tissue in knee joints. Significantly improved macroscopic observation and histological score were noted in the MSCs-PCC group, which achieved sufficient cartilaginous repair and regeneration. The MSCs coated with PCC may have a promising prospect for clinical applications in cartilage repair and regeneration.

## Additional files

Additional file 1: **Figure S1.** Concentration of residual collagen I in MSC suspension after PCC. Concentration of col. I detected (*n* = 5) by Rat Collagen I ELISA Kit (LSBio, USA). (JPG 133 kb)

Additional file 2: **Figure S2.** Ex-vivo adhesion of MSCs suspended in col. I solution. BLIS analysis of MSCs suspended in 10 ng/ml, 500 ng/ml, and 10μg/ml col. I solution on cartilage slices. MSCs pretransfected by luciferase and the total adhered MSC number calculated by luminescent intensity (*n* = 5 in each group). (JPG 86 kb)

Additional file 3: **Figure S3.** In-vivo homing suspended in col. I solution. **a, b** BLIS analysis of luminescent distribution within left knees of New Zealand rabbits at 2 days after DIAI of luciferase-labeled MSCs or MSCs suspended in 10 ng/ml col. I solution. Red frames indicate cartilage defect (**a**, *n* = 3 in each group). Luminescent intensity of total ROI radiance within cartilage defect (red frame) calculated to reveal homing of MSCs (**b**). **a** Sagittal frozen sections of cartilage lesion site, red particles are CM-DiI-labeled MSCs (scale bar, 400 μm). (TIF 1614 kb)

## Abbreviations

AFM: Atomic force microscope
BLIS: Bioluminescent imaging system
BSA: Bovine serum albumin
CFU-F: Colony-forming unit fibroblasts
CM-DiI: 1,1′-Dioctadecyl-3,3,3′,3′-tetramethylindocarbocyanine perchlorate
col. I: Type I collagen
col. II: Type II collagen
col. X: Type X collagen
DAPI: 4,6-Diamino-2-phenyl indole
DIAI: Direct intra-articular injection
DMEM: Dulbecco’s modified Eagle’s medium
FITC: Fluorescein isothiocyanate
FN: Fibronectin
GAPDH: Glyceraldehyde-3-phosphate dehydrogenase
ICRS: International Cartilage Repair Society
ITG: Integrin
LSCM: Laser scanning confocal microscope
MSC: Mesenchymal stem cell
N-CAM: Neural cell adhesion molecule
OA: Osteoarthritis
PBS: Phosphate buffer saline
PCC: Pericellular collagen I coating
PCM: Pericellular matrix
RT-qPCR: Real time-quantitative polymerase chain reaction
SEM: Scanning electron microscope
SOX-9: Sry related HMG box-9
