# Supplementary figures and images for: Pericellular collagen I coating for enhanced homing and chondrogenic differentiation of mesenchymal stem cells in direct intra-articular injection
Source: Stem Cell Res Ther. 2018 Jun 27;9:174. doi: 10.1186/s13287-018-0916-z (PMC6020325; doi:10.1186/s13287-018-0916-z)

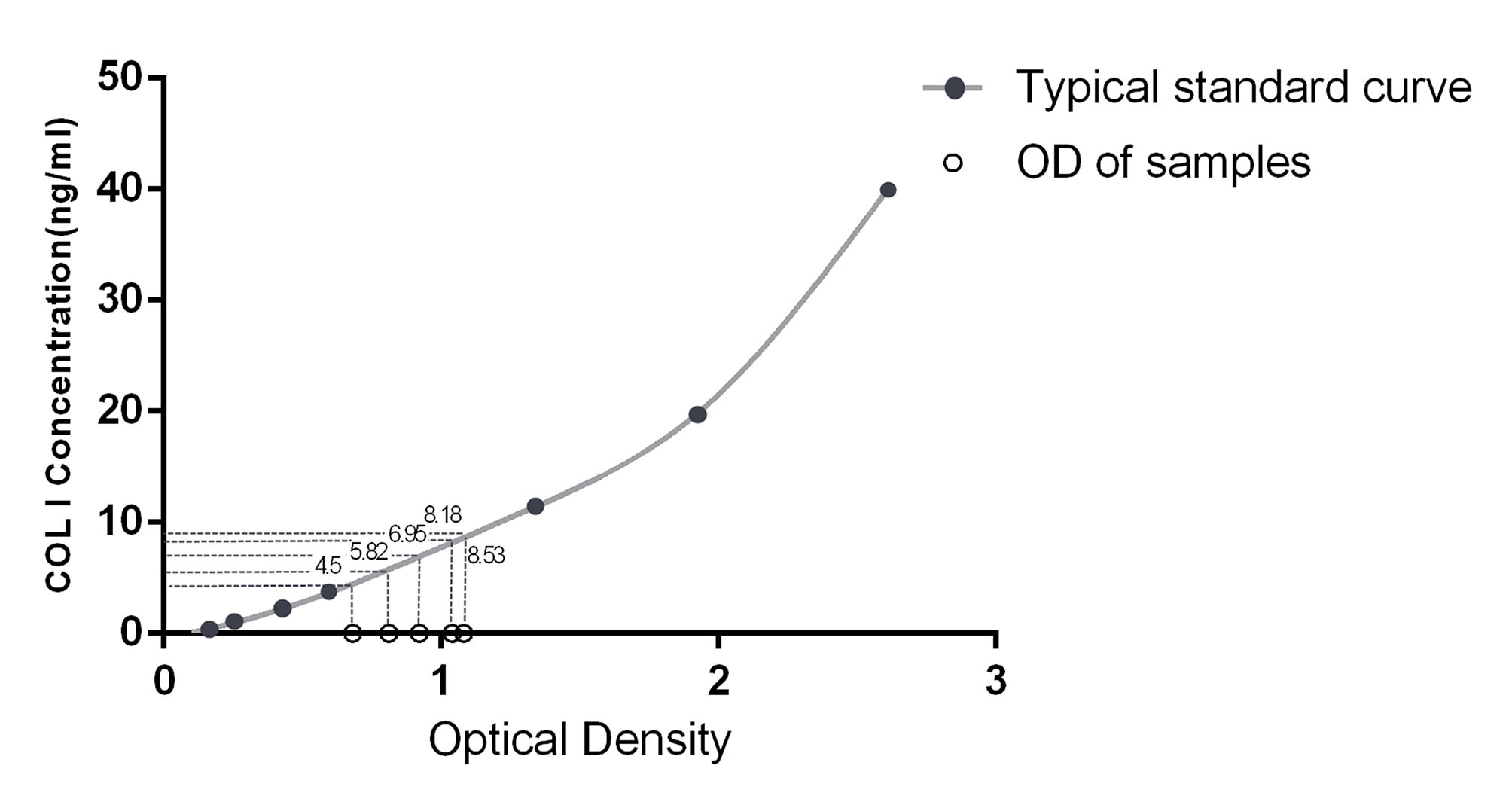

Supplement: Supplementary file 1 — Figure S1. Concentration of residual collagen I in MSC suspension after PCC. Concentration of col. I detected (n = 5) by Rat Collagen I ELISA Kit (LSBio, USA). (JPG 133 kb) [file 13287_2018_916_MOESM1_ESM.jpg]

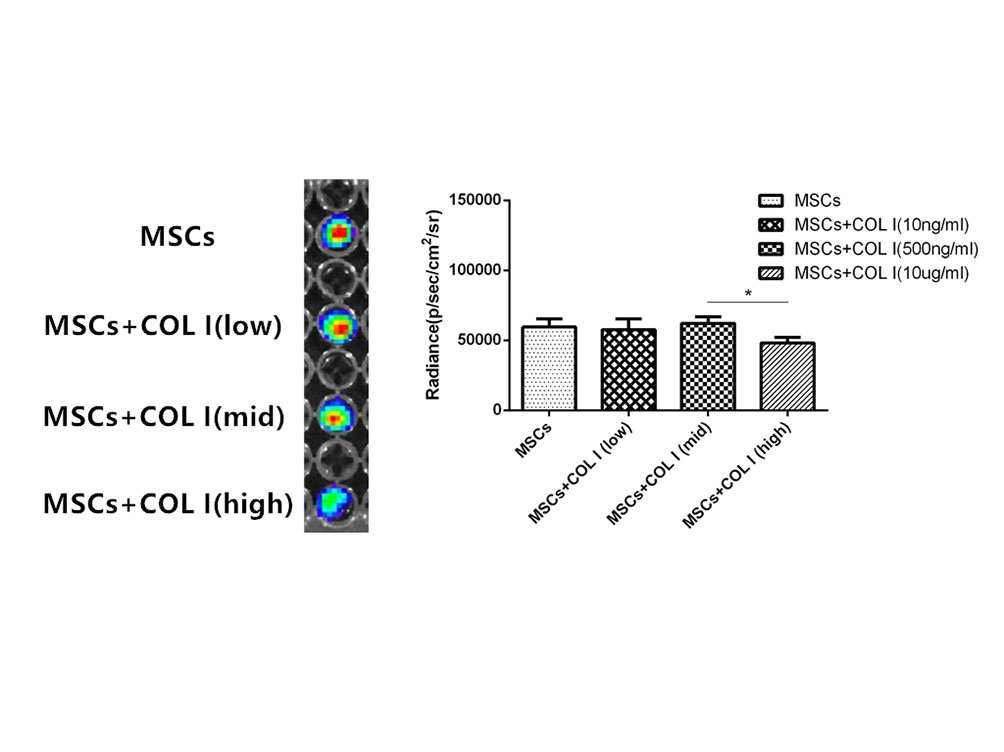

Supplement: Supplementary file 2 — Figure S2. Ex-vivo adhesion of MSCs suspended in col. I solution. BLIS analysis of MSCs suspended in 10 ng/ml, 500 ng/ml, and 10μg/ml col. I solution on cartilage slices. MSCs pretransfected by luciferase and the total adhered MSC number calculated by luminescent intensity (n = 5 in each group). (JPG 86 kb) [file 13287_2018_916_MOESM2_ESM.jpg]

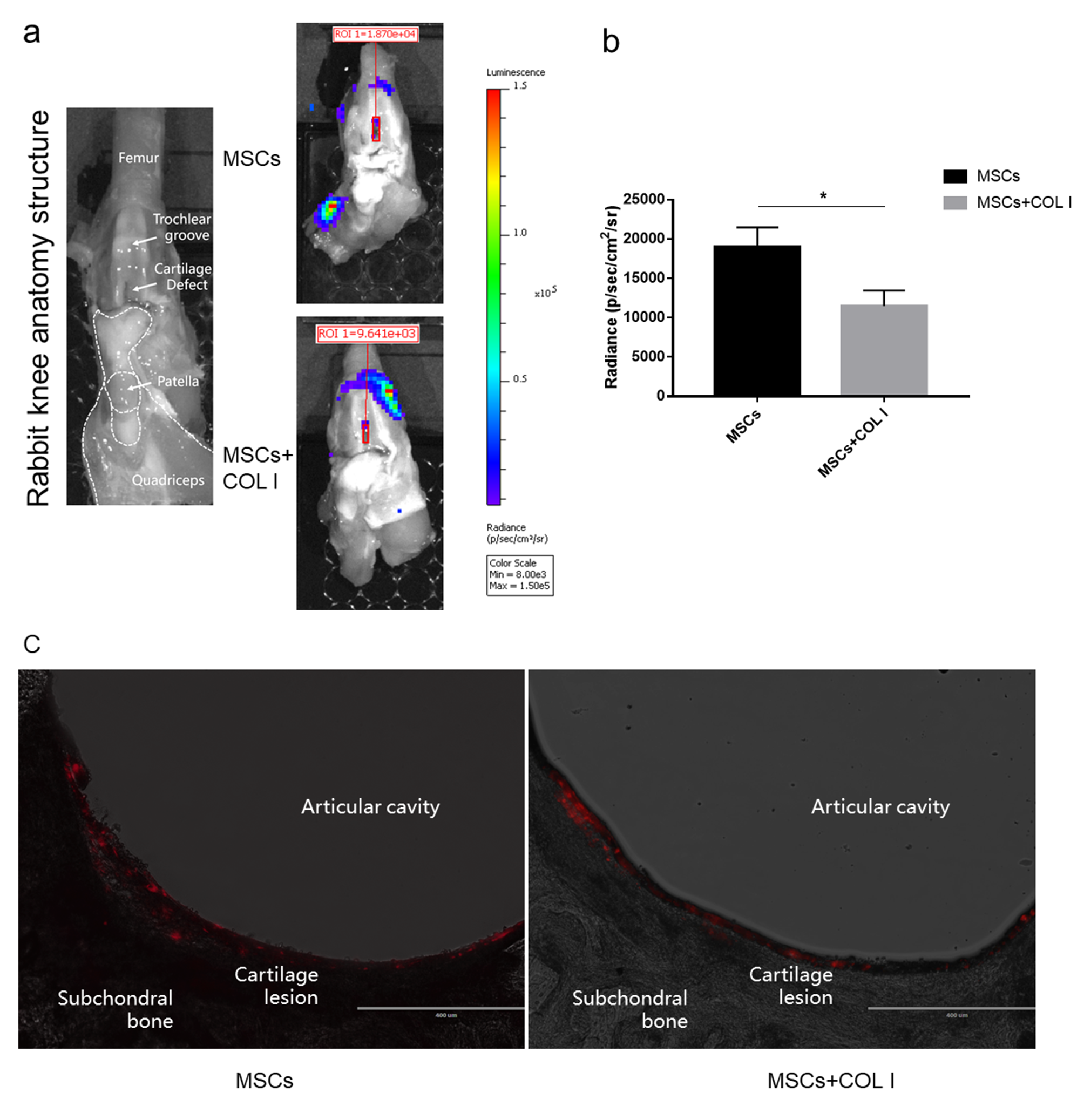

Supplement: Supplementary file 3 — Figure S3. In-vivo homing suspended in col. I solution. a, b BLIS analysis of luminescent distribution within left knees of New Zealand rabbits at 2 days after DIAI of luciferase-labeled MSCs or MSCs suspended in 10 ng/ml col. I solution. Red frames indicate cartilage defect (a, n = 3 in each group). Luminescent intensity of total ROI radiance within cartilage defect (red frame) calculated to reveal homing of MSCs (b). a Sagittal frozen sections of cartilage lesion site, red particles are CM-DiI-labeled MSCs (scale bar, 400 μm). (TIF 1614 kb) [file 13287_2018_916_MOESM3_ESM.tif]
